# Metronidazole-induced encephalopathy delayed diagnosis due to cerebellar infarction: A case report

**DOI:** 10.1097/MD.0000000000032788

**Published:** 2023-02-03

**Authors:** Sangha Cha, Byung Chan Lee, Chang-Won Moon, Kang Hee Cho

**Affiliations:** a Department of Rehabilitation Medicine, School of Medicine, Chungnam National University, Daejeon, Korea; b Department of Biomedical Institute, Chungnam National University, Daejeon, Korea.

**Keywords:** drug side effect, metronidazole, metronidazole-induced encephalopathy

## Abstract

Metronidazole is a 5-nitroimidazole antibiotic effective against anaerobic bacterial and parasitic infections. Long-term use may cause side effects in the central nervous system, although the occurrence of encephalopathy is rare.

**Patient concerns:**

A 73-year-old man was diagnosed with acute pyelonephritis and received antibiotic treatment. During the treatment, the patient complained of back pain. Lumbar spinal magnetic resonance imaging (MRI) revealed infective spondylitis, and metronidazole (1.5 g) was administered daily for approximately 160 days. The patient developed cognitive dysfunction and gait disorder after antibiotic treatment, and brain MRI showed acute infarction in both cerebellar lobes. Secondary prevention with antiplatelet and physiotherapy was prescribed; however, functional recovery was not achieved.

**Diagnosis:**

After 1 month, a follow-up brain MRI showed high signal intensity and diffusion restriction in the corpus callosum on diffusion-weighted images and high signal intensity in the dentate nucleus on T2-weighted images. Therefore, metronidazole-induced encephalopathy was suspected.

**Interventions:**

Metronidazole was discontinued, and ceftriaxone (2 g/day) was administered to manage the infective spondylitis.

**Outcomes:**

One week after the discontinuation of the drug, the patient’s cognition improved to the extent that communication was possible. Thus, even if other neurological deficits, such as cerebellar infarction, are found in patients with long-term disability, the possibility of metronidazole-induced encephalopathy should be considered when metronidazole is used for a long time.

## 1. Introduction

Metronidazole is a 5-nitroimidazole antibiotic effective against anaerobic bacterial and parasitic infections. Its common side effects include nausea (12%–64%), and it has a bitter taste (65%–72%).^[1]^ Long-term use of metronidazole may cause adverse effects on the nervous system, such as metronidazole-induced encephalopathy (MIE).^[2]^ Because MIE is not a commonly reported case, its diagnosis could be easily overlooked. If other illnesses are present simultaneously, diagnosis can be delayed, making it challenging to take necessary action. To the best of our knowledge, there are no cases in which the diagnosis of MIE was delayed due to a preceding stroke. Here, we report the case of a patient initially diagnosed with cerebellar infarction and later diagnosed with MIE due to the long-term use of metronidazole for infective spondylitis, along with a literature review.

## 2. Case presentation

**Figure 1.**
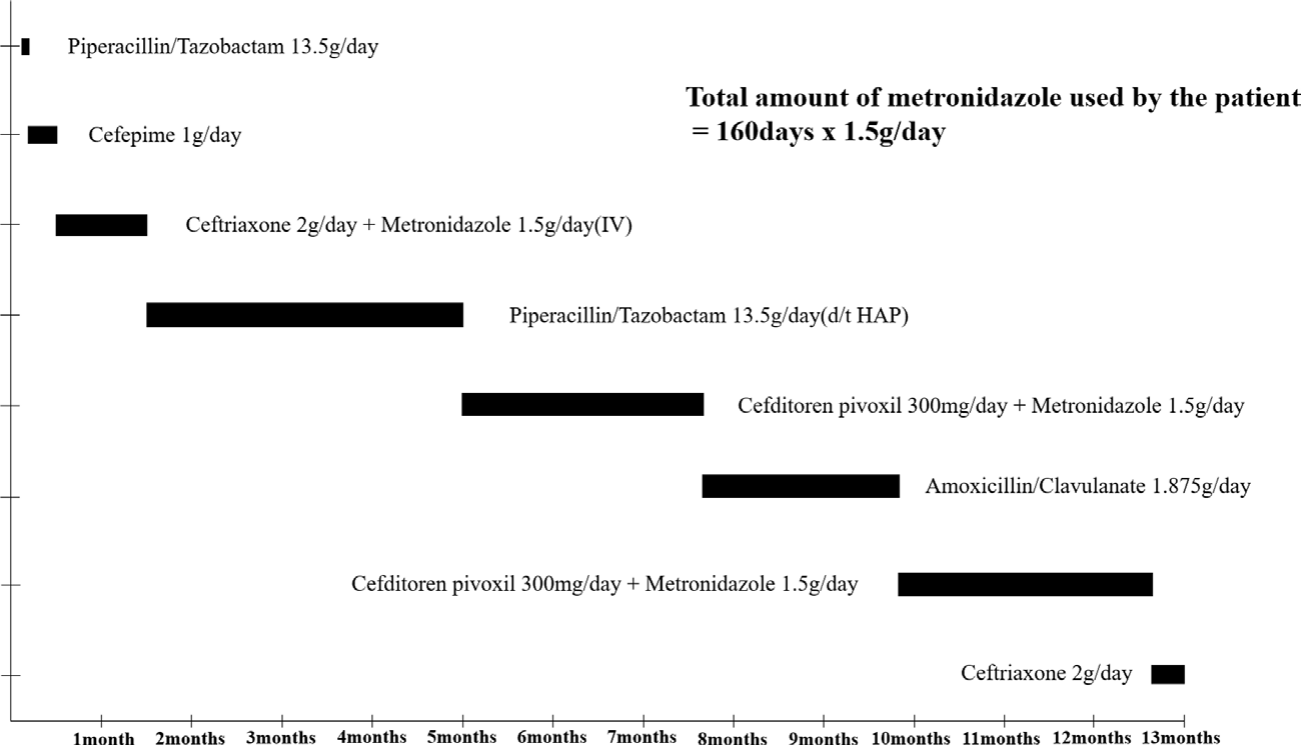
Timeline of antibiotics prescribed to the patient.

**Figure 2.**
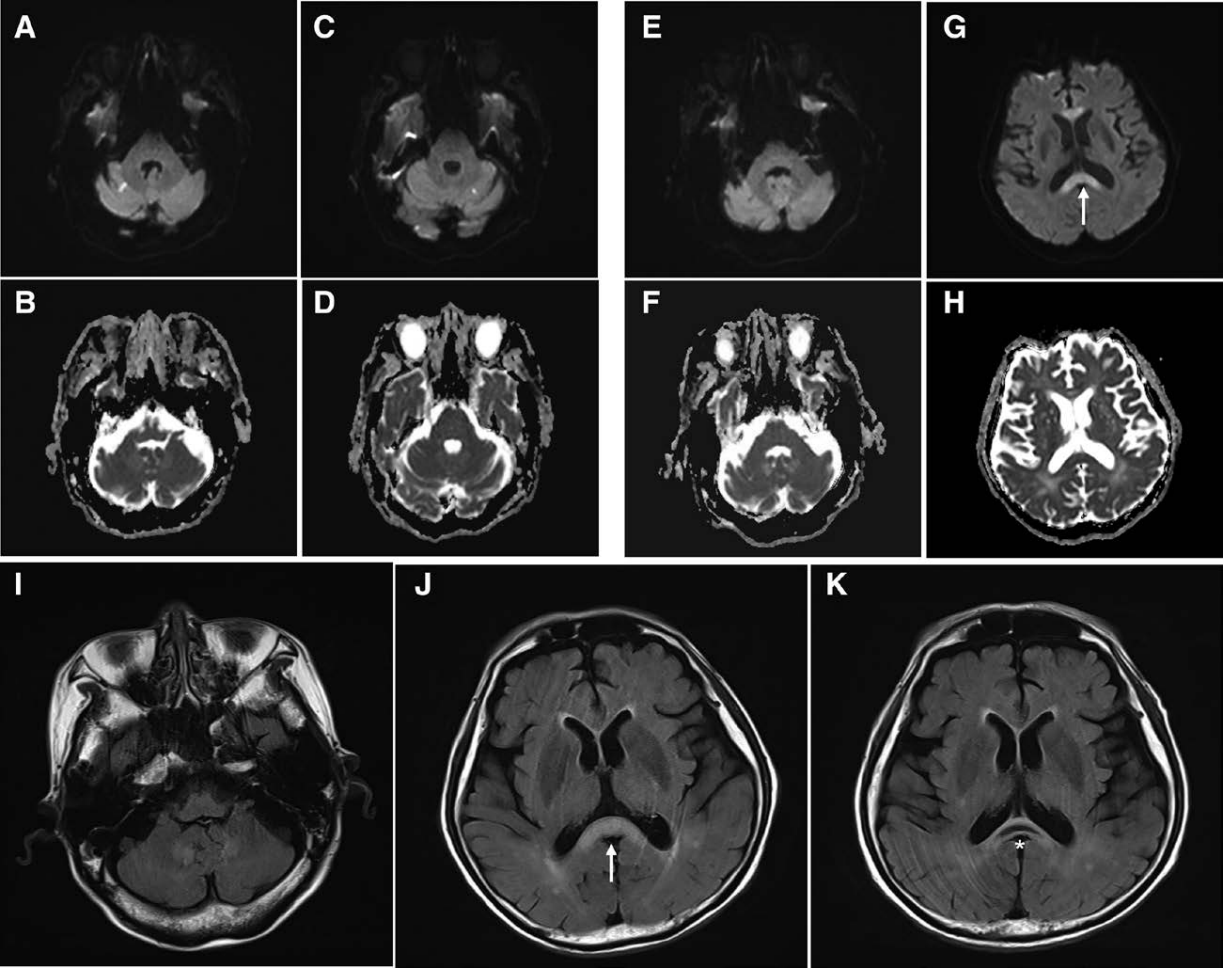
Initially obtained brain MRI of the patient showed an acute ischemic stroke in the bilateral cerebellar hemisphere (A, B, C, D). One month after the initial brain MRI, a follow-up brain MRI was performed, which showed diffusion restriction in DWI images in the corpus callosum (white arrow), but no abnormal signals in both dentate nuclei (E, F, G, H). In T2-weighted FLAIR images, the high signal intensity of the corpus callosum was depicted (white arrow, I, J), and the second follow-up MRI showed encephalomalacia in the corpus callosum (asterisk, K). DWI = diffusion-weighted images, FLAIR = fluid-attenuated inversion recovery, MRI = magnetic resonance imaging.

A 73-year-old man presented to the emergency room with a fever and acute pyelonephritis. The fever subsided after antibiotic treatment; however, the patient complained of severe back pain. Magnetic resonance imaging (MRI) of the spine with contrast was performed, and the patient was diagnosed with infective spondylitis. Surgical treatment (L3-4-5 laminectomy, fusion, and abscess drainage) was performed. Subsequently, he was transferred to the Department of Rehabilitation Medicine. At the time of transfer, the strength of the upper and lower extremities was generally evaluated as grade 4 on the medical research council scale. The patient was able to walk independently using a walking aid. Antibiotic treatment was continued, and metronidazole was taken at 1.5 g/day for approximately 160 days (Fig. 1). After 9 weeks of physiotherapy, he was discharged home; however, 1 month after discharge, he was re-hospitalized because of persistent physical and cognitive dysfunction deterioration. In the manual muscle test, the patient’s muscle strength in both the upper and lower extremities decreased from 4 to 2 on the medical research council scale. In addition, the berg balance test score decreased from 40 to 5 points, and cognitive function using the korean-mini mental state examination decreased from 26 to 20 points compared with initial admission. The Korean Modified Barthel Index test showed that the patient had more difficulty in performing activities of daily living, which was reduced from 67 to 30 points compared with that 1 month ago. Brain MRI was performed, and an ischemic stroke was diagnosed in both lobes of the cerebellum (Fig. 2A–D). However, the patient suffered from continuous deterioration of cognitive and physical functions even after the diagnosis of ischemic stroke. Therefore, a follow-up brain MRI was performed 2 months after the initial MRI, and it showed an increased signal in the cerebellar dentate nucleus and corpus callosum on T2-weighted fluid-attenuated inversion recovery (FLAIR) images, diffusion restrictions in diffusion-weighted images (DWI), and no significant changes in apparent diffusion coefficient maps in the corpus callosum, suggesting the occurrence of cytotoxic edema in the affected area rather than ischemic stroke (Fig. 2E–J). Therefore, combining these findings and the medical history, MIE had been highly suspected. Metronidazole was discontinued, and 2 g of ceftriaxone daily was prescribed to manage the patient infective spondylitis. One week after the discontinuation of metronidazole, the patient cognitive function gradually improved, and his mini-mental state examination score increased from 20 to 25 points. One month after discontinuation of metronidazole, a follow-up brain MRI showed encephalomalacia in the corpus callosum on T2-weighted FLAIR images (Fig. 2K), and abnormal signal intensity in both the dentate nuclei was diminished.

## 3. Discussion

In this report, we present a case in which MIE was found because of the persistent decline in cognition and motor functions of the patient after a stroke. Stroke is common in the elderly and may cause deterioration in cognitive and motor functions; however, it is not uncommon to show deterioration of these functions continuously after the first onset. Although this continued deterioration of function was shown by overlapping MIEs, it is rare for the diagnosis to be delayed because of the effects of a previous stroke.

Metronidazole is a broad-spectrum antibiotic used worldwide to treat infections caused by anaerobic bacteria, protozoa, and ameba. It is relatively safe and does not have many side effects; however, side effects, such as dizziness, ataxia, dysarthria, peripheral neuropathy, convulsions, and encephalopathy, are observed.^[3]^ In 138 cases of MIE reported in 2020, men (n = 88, 65%) were more affected than women (n = 48, 35%), and the average age of adults (130/138) was 56.8 years.^[4]^ The duration and indication for metronidazole use varied depending on each patient and report. However, the average period was 101.6 days, ranging from 2 days to almost 8 years, the cumulative dose varied from 5 g to 2000 g, and an average of 125.7 g was used as the cumulative dose.^[4]^ In our case, the patient was prescribed metronidazole 1.5 g/day for approximately 160 days, and the total cumulative dose was 240 g (Fig. 1).

The pathophysiology of MIE has not been elucidated; however, several mechanisms have been proposed, suggesting that metronidazole may reduce the catecholamine neurotransmitter, which may lead to the formation of free radicals in the process, resulting in neurotoxicity.^[3,5]^ MIE can present with dysarthria (63%), gait instability (55%), limb dyscoordination (53%), altered mental status (41%), and dizziness (18%).^[4]^ However, no clear pathognomonic signs or symptoms were observed in the current case. In addition, vague symptoms and the chronic progressive nature of the disease could be the cause of delayed diagnosis. The findings of the bilateral cerebellar infarction on the first brain MRI appear to be incidental. Furthermore, in our case, the diagnosis was delayed mainly because of the presence of mild cerebellar stroke, which also presented with mild dysarthria and gait disturbance.

MRI is the most useful diagnostic tool for these patients who have neurological changes The most characteristic imaging findings of MIE are symmetric hyperintensity of the cerebellar dentate nuclei, splenium of the mesocerebrum, dorsal pontine, and corpus callosum on T2-weighted and FLAIR images.^[4,6,7]^ In addition, among several MIE cases, high signal intensity on the DWI of these structures indicates metronidazole-induced cytotoxicity and angioedema.^[8]^ Due to the persistent neurological deterioration in our case, we performed a follow-up MRI, which showed high signal intensity on T2 FLAIR images in the corpus callosum and dentate nucleus, and further diffusion restriction on DWI in the corpus callosum was observed, leading to the final diagnosis of MIE.

The treatment of MIE involves the discontinuation of metronidazole and conservative supportive management. Most patients with MIE have a favorable prognosis after the discontinuation of metronidazole treatment. Most patients either fully recover (29%) or improve their symptoms (65%).^[8]^ In most cases, symptoms and follow-up imaging findings were recovered from the initial images. However, some patients had persistent neurological sequelae, especially when cystic necrotic degeneration developed on follow-up MRI.^[3,5,7,9]^ In our patient, cystic necrotic degeneration appeared in the corpus callosum, with a poor prognosis. The cause of the poor prognosis could be irreversible damage to necrotic brain tissues, mostly due to late detection and prolonged high-dose metronidazole use.

Herein, we present a case report of a late diagnosis of MIE due to mild cerebellar infarction. Suspicion from the physician and close patient monitoring could be the key to diagnosing MIE. Moreover, in patients using metronidazole with chronic progressive functional loss, even with proper care and rehabilitation therapy, MIE should be suspected, and a brain MRI should be performed.

## Acknowledgements

The authors would like to thank the patient for his contributions to this work and Editage (www.editage.co.kr) for editing and reviewing this manuscript in English.

## Author contributions

**Conceptualization:** Sang Ha Cha, Byung Chan Lee, Chang Won Moon.

**Data curation:** Sang Ha Cha, Byung Chan Lee, Chang Won Moon.

**Supervision:** Kang Hee Cho.

**Writing – original draft:** Sang Ha Cha.

**Writing – review &amp; editing:** Sang Ha Cha, Byung Chan Lee, Chang Won Moon.
